# Application of Artificial Intelligence in Sickle Cell Identification From Blood Smears: A Potential Game Changer for Developing Nations

**DOI:** 10.7759/cureus.95563

**Published:** 2025-10-28

**Authors:** Puja Singh, Mitesh Shah, Ashish K Dubey

**Affiliations:** 1 Pathology, Jawaharlal Nehru Medical College, Wardha, IND; 2 Pathology, Bundelkhand Medical College, Sagar, IND; 3 Surgery, Bundelkhand Medical College, Sagar, IND

**Keywords:** artificial intelligence in medicine, deep learning systems, hemoglobinopathy, machine learning healthcare data, sickle cell disease

## Abstract

Background

Sickle cell disease (SCD) is a genetic disorder affecting hemoglobin, leading to blood flow blockage and symptoms like pain and organ failure. It poses a significant global health burden, especially in regions such as sub-Saharan Africa and India. Early diagnosis is vital, but it is often hindered by traditional methods that require specialized resources. Artificial Intelligence (AI) is emerging as a solution, enhancing diagnostic techniques through faster and more accurate identification of SCD in blood smears, ultimately improving patient outcomes. This study focuses on developing AI applications to improve the early detection of SCD.

Methods

This study involved 81 participants. Of these, eight cases with thalassemia, hemoglobin E disease (HbE), and hemoglobin D disease (HbD) diagnoses were excluded from the study. The remaining 73, comprising 13 negative and 60 with sickle cell anemia (SS), sickle cell trait (AS), and AS+thalassemia, were included in the study. Each participant's blood sample underwent complete blood count (CBC), peripheral smear, and hemoglobin electrophoresis tests, with 730 data points generated from captured images analyzed by trained pathologists. The diagnosis by hemoglobin electrophoresis served as the gold standard, categorizing SCD as 1 and normal cells as 0. AI algorithms, including GoogLeNet and ResNet models, were developed using Python (Python Software Foundation, Delaware, United States) in Google Colab (Google LLC, Mountain View, California, United States), with performance assessed using sensitivity, specificity, recall, and F1-score metrics.

Results

Demographic data from participants indicates that the majority were aged 18-30, with 42 (57.53%) male participants. Analysis of CBC parameters revealed significant differences in hemoglobin, mean corpuscular volume (MCV), and mean corpuscular hemoglobin (MCH) between normal and SCD patients. Of those tested with hemoglobin electrophoresis, 13 (16.05%) were negative, while 60 (74.08%) tested positive for SCD, excluding cases with thalassemia, HbE, and HbD for AI analysis. A confusion matrix was used to assess the classification model's performance, focusing on true positives and negatives, as well as errors. Performance metrics such as accuracy, precision, recall, sensitivity, specificity, and F1-score were reported for three AI models, with ResNet50 convolutional neural network achieving the highest performance, followed by GoogLeNet and ResNet18.

Conclusion

This study confirms the high accuracy of AI in identifying sickle cells in blood smears. Despite challenges in validation, infrastructure, and adoption, AI-assisted screening could reduce diagnostic delays and improve outcomes in regions heavily impacted by SCD.

## Introduction

Sickle cell disease (SCD) is a genetic disorder affecting hemoglobin, the protein responsible for oxygen transport in red blood cells [1]. This condition leads to blood flow blockage and various clinical manifestations such as vaso-occlusion, chronic pain, and organ failure [1]. SCD has a significant global health burden. In 2021, the Global Burden of Disease (GBD) study estimated the all-age rate for SCD in India was 89.6 (95%CI: 66.3-117) per 100,000 population [2]. SCD is particularly prevalent in sub-Saharan Africa, India, Saudi Arabia, and Mediterranean countries [1].

Early diagnosis of SCD is crucial to reducing patient morbidity and mortality. Traditional diagnostic procedures, blood smear examination, hemoglobin electrophoresis, and genetic testing require specialized professionals and laboratory infrastructure. These limitations cause delayed results, misinterpretation, and accessibility issues, particularly in resource-limited settings [1,3].

This gap in healthcare access and quality can potentially be alleviated by artificial intelligence (AI) [4]. AI is rapidly transforming multiple domains in healthcare, revolutionizing diagnostic techniques. It can offer faster and more accurate identification of SCD from blood smears [5]. The integration of sophisticated image processing techniques, machine learning (ML), and deep learning (DL) algorithms into diagnostic workflows can significantly enhance diagnostic speed and precision, ultimately transforming SCD management and improving patient outcomes.

This study aims to develop and evaluate the application of AI in the identification of SCD from blood smears, enhancing the accuracy, efficiency, and accessibility of early detection.

## Materials and methods

This prospective, cross-sectional, and comparative study was conducted at Bundelkhand Government Medical College, Sagar, Madhya Pradesh, India, from March 15, 2023, to January 31, 2025. The study was approved by the Institutional Ethics Committee, Bundelkhand Medical College (approval number: 223/IECBMC/2023, dated March 10, 2023).

Study population

A total of 81 patients, over 18 years, who were advised for sickling or peripheral smear (PS) (using Leishman stain) examination were included in the study. Patients previously diagnosed with other hemoglobinopathies were excluded (eight cases) from AI analysis. Written consent was obtained from all study participants.

Procedure

From each participant, 2 mL of blood was drawn in an ethylenediaminetetraacetic acid (EDTA) vial. Complete blood count (CBC), PS, and hemoglobin electrophoresis tests were performed on this blood. Ten pictures, from different regions, of each PS, at 100x resolution, were captured, creating a total of 730 data points. Sample size for confidence level (95%), expected accuracy (p=0.95), and margin of error (±2%) comes out to be 456. Data points for this study are way higher than this figure. These images were then labeled by two trained pathologists using LabelImg by Tzutalin (released in 2015; https://github.com/HumanSignal/labelImg) and then fed into different pretrained AI algorithms to make a diagnosis.

Pre-trained AI algorithms used in this study were ResNet50 (https://docs.pytorch.org/vision/main/models/generated/torchvision.models.resnet50.html), ResNet18 (https://pytorch.org/vision/main/models/generated/torchvision.models.resnet18.html), and GoogLeNet (https://pytorch.org/hub/pytorch_vision_googlenet/). Residual networks, ResNet18 and ResNet50, are excellent for learning subtle morphological features (e.g., sickle shape, texture, and contour differences). ResNet18 uses BasicBlock for shallow and fast processing, and ResNet50 uses Bottleneck Block for efficiency in deeper layers. GoogLeNet enables multi-scale feature extraction, helpful in identifying cells of varying shapes and sizes. It is efficient with fewer parameters compared to deep ResNets. Key parameters used for these algorithms are presented in Table 1.

**Table 1:** Key parameters used for artificial intelligence algorithms

Model	Epochs	Batch Size	Optimizer	Learning Rate	Weight Decay
ResNet18	75	32	Adam	0.0003	1.00E-05
GoogLeNet	80	32	Adam	0.0005	1.00E-05
ResNet50	150	32	SGD (momentum=0.9)	0.001	1.00E-04

Diagnosis made by haemoglobin electrophoresis, using the AELAB AE-SubMINI Horizontal Electrophoresis Apparatus (AELAB GUANGZHOU Co. Ltd, Guangdong, China), was considered the gold standard. SCD were labeled as 1, and normal cells were labeled as 0. Implementation was performed in Google Colab (Google LLC, Mountain View, California, United States) to speed up the training and inference processes significantly. The artificial intelligence algorithms were developed using Python (v3.9; Python Software Foundation, Delaware, United States). Data distribution was analyzed using percentages, means, and standard deviations. Sensitivity, specificity, recall, and F1-score were calculated to evaluate the performance of different AI algorithms.

## Results

The gold standard test, hemoglobin electrophoresis, was performed for all participants. Of the 81 participants, 13 (16.05%) were negative, and 60 (74.08%) were SCD positive. The eight cases with thalassemia, hemoglobin E disease (HbE), and hemoglobin D disease (HbD) diagnoses were excluded from AI analysis. The distribution of diagnoses made by the gold standard is presented in Table 2.

**Table 2:** Distribution of diagnoses made by hemoglobin electrophoresis (N =81) NOTE: SCD positive cases belong to categories: AS, SS, or AS+Thalassemia AS: sickle cell trait; SS: sickle cell anemia; HbE: hemoglobin E disease; HbD: hemoglobin D disease; SCD: sickle cell disease

Diagnosis	Count	Percentage
Normal	13	16.05
AS	33	40.74
SS	10	12.35
AS+thalassemia	17	20.99
Thalassemia	6	7.41
HbE	1	1.23
HbD	1	1.23

The demographic distribution of participants, based on age and gender, is presented in Table 3, along with the distribution of key CBC parameters. Most participants were in the 18-30 age group, and 42 (57.53%) were male. It is to be noted that the eight excluded cases were not included in the analysis from here on.

**Table 3:** Demographic distribution and distribution of relevant CBC parameters (N=73) CBC: complete blood count

Group	Count	Percentage
Age (in years)		
18-30	53	72.6
31-45	13	17.81
46-60	7	9.59
Gender		
Male	42	57.53
Female	31	42.47

Significant variations in parameters Hb, MCV, and MCH were observed between normal and SCD patients. However, no significant difference, with a p-value of 0.1043> 0.05, was observed for MCHC, as presented in Table 4.

**Table 4:** Distribution of relevant CBC parameters (N=73) SCD: sickle cell disease; CBC: complete blood count; MCV: mean corpuscular volume; MCH: mean corpuscular hemoglobin; MCHC: mean corpuscular hemoglobin concentration; Hb: hemoglobin * p-values are calculated based chi-square test

Parameters (Unit)	Normal (n=13), mean±SD	SCD (n=60), mean±SD	p-value*
Hb (G/DL)	12.75 ± 0.65	4.62 ± 1.29	< 0.0001
MCV (fL)	91.46 ± 3.67	74.67 ± 2.34	< 0.0001
MCH (PG)	29.61 ± 1.28	24.39 ± 1.33	< 0.0001
MCHC (G/DL)	33.75 ± 1.22	34.26 ± 2.37	0.1043

**Figure 1:**
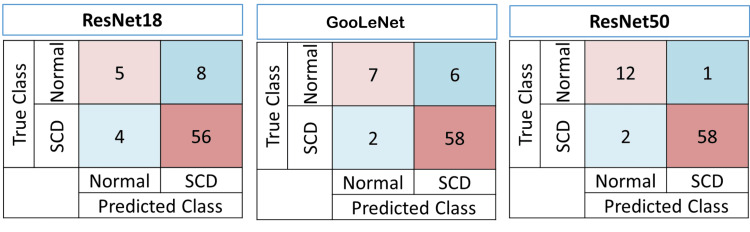
Confusion matrices for different artificial intelligence algorithms: ResNet18, GoogLeNet, and ResNet50 (N=73) SCD: sickle cell disease

The confusion matrix presented in Figure 1 was used to perform a quantitative analysis of the classification model's performance. True positives and true negatives are crucial indicators of performance. Errors, false positives, and false negatives are crucial for refining the models.

Table 5 presents the key performance parameters for these three AI network models, including accuracy, precision, recall, sensitivity, specificity, and F1-score. Overall, ResNet50 convolutional neural networks (CNNs) performed best, followed by GoogLeNet and ResNet18.

**Table 5:** Performance parameters of different artificial intelligence algorithms

Algorithm	Accuracy (%)	Precision	Recall	Sensitivity	Specificity	F1-Score
ResNet18	83.56%	0.875	0.9333	0.9333	0.3846	0.9032
GoogLeNet	89.04%	0.9063	0.9667	0.9667	0.5385	0.9355
ResNet50	95.90%	0.983	0.9667	0.9667	0.923	0.9748

## Discussion

The application of AI in sickle cell identification from blood smears presents a transformative opportunity, particularly for developing nations where the disease burden is high and diagnostic resources are often limited. This study investigates the performance of various AI algorithms in classifying sickle cells. 

​The demographic composition of our dataset was designed to reflect the heterogeneous populations found in high-SCD-prevalence regions, including variations in age and sex. This mitigated algorithmic bias, and coverage of morphological variations in red blood cells can be influenced by age and comorbid conditions (e.g., malaria, nutritional deficiencies) common in developing nations [6]. However, patients younger than 18 years were excluded from the study.

​ML algorithms have shown remarkable success in diagnosing SCD using blood smear images and hematology data ​[1]. CNNs have consistently demonstrated superior accuracy compared to other methods. ​Studies have reported accuracies for DL models reaching up to 100% on specific metrics. For example, the ResNet-50 algorithm achieved 100% precision, recall, and F1-score for classifying circular, elongated, and other cell shapes ​[1]. ​Another study utilizing a smartphone microscope and DL achieved 98% accuracy and an area under the curve (AUC) of 0.99 for sickle cell identification [7]. ​Hybrid recurrent neural network (RNN) models with optimization techniques have also reported high accuracies of 99.8%, precision of 99.7%, recall of 98.4%, and F1-score of 98.5%. ​Even traditional algorithms like Multilayer Perceptron, with robust clinical data, can achieve comparable performance, with one study reporting 99.0% accuracy. In this study, ResNet50 performed best with accuracy, precision, recall, and F1-score as 95.90%, 0.983, 0.9667, and 0.9748, respectively. It was followed by GoogleNet and ResNet18. Slightly low accuracy could be fewer cases included in the study.

The implementation of AI-enabled tools for blood smear pre-screening offers several significant advantages, especially for developing nations. ​These tools can interpret high-throughput screening results with increased precision, leading to reduced diagnostic turnaround times [8]. ​This automation helps overcome challenges related to human error, subjectivity, and the time-consuming nature of manual examination by expert professionals [9,10]. ​Smartphone-based microscopy, coupled with DL, provides a mobile, cost-effective, and accurate method for SCD screening, increasing practicality in low-resource settings [11]. ​Such systems can classify various SCD profiles with high accuracy, often exceeding 98%, and can even assign probability values to classifications, empowering clinicians with critical information for informed decisions [12]. The ability to analyze images rapidly (e.g., six minutes per image for AI versus up to two hours for manual classification) and consistently, with significantly lower uncertainty (3% for ML vs. ​20% for human counts), underscores the efficiency and reliability of AI tools [13].

Despite the promising results, several challenges impede widespread adoption. The "black box" nature of some complex models can erode clinician trust; efforts in explainable AI (XAI), such as generating heatmaps to highlight decisive cellular features, are essential to bridge this gap [14]. Infrastructure hurdles, including reliable electricity and internet connectivity for cloud-based models, are non-trivial in many target regions. Developing robust, lightweight models capable of offline operation is a key research direction. Furthermore, regulatory approval and integration into existing clinical workflows present significant hurdles. Continuous validation on larger, prospective datasets from diverse geographical locations is required to meet regulatory standards and prove real-world efficacy [9,15].

The following limitations should be considered for this study. The study is based on a small sample size, which can restrict the generalizability of its findings. ​Data for the age group less than 18 should be included. Absence of external validation can compromise the reproducibility of results in different populations. ​

## Conclusions

This study confirms AI's high accuracy in identifying sickle cells from blood smears. While challenges in validation, infrastructure, and adoption remain, AI-assisted screening holds promise to reduce diagnostic delays, optimize resource use, and significantly improve outcomes in regions disproportionately burdened by SCD. Future work must focus on robust, field-deployable systems and prospective validation to fully realize this potential and improve early diagnosis outcomes globally.
